# A Randomized Controlled Trial Translating the Diabetes Prevention Program to a University Worksite, Ohio, 2012–2014

**DOI:** 10.5888/pcd12.150301

**Published:** 2015-11-25

**Authors:** Kellie R. Weinhold, Carla K. Miller, David G. Marrero, Haikady N. Nagaraja, Brian C. Focht, Gregg M. Gascon

**Affiliations:** Author Affiliations: Kellie R. Weinhold, Haikady N. Nagaraja, Brian C. Focht, The Ohio State University, Columbus, Ohio; David G. Marrero, Indiana University School of Medicine, Indianapolis, Indiana; Gregg M. Gascon, The Ohio State University Health Plan, Inc, Columbus, Ohio.

## Abstract

**Introduction:**

Working adults spend much time at the workplace, an ideal setting for wellness programs targeting weight loss and disease prevention. Few randomized trials have evaluated the efficacy of worksite diabetes prevention programs. This study evaluated the efficacy of a worksite lifestyle intervention on metabolic and behavioral risk factors compared with usual care.

**Methods:**

A pretest–posttest control group design with 3-month follow-up was used. Participants with prediabetes were recruited from a university worksite and randomized to receive a 16-week lifestyle intervention (n = 35) or usual care (n = 34). Participants were evaluated at baseline, postintervention, and 3-month follow-up. Dietary intake was measured by a food frequency questionnaire and level of physical activity by accelerometers. Repeated measures analysis of variance compared the change in outcomes between and within groups.

**Results:**

Mean (standard error [SE]) weight loss was greater in the intervention (−5.5% [0.6%]) than in the control (−0.4% [0.5%]) group (*P* < .001) postintervention and was sustained at 3-month follow-up (*P* < .001). Mean (SE) reductions in fasting glucose were greater in the intervention (−8.6 [1.6] mg/dL) than in the control (−3.7 [1.6] mg/dL) group (*P* = .02) postintervention; both groups had significant glucose reductions at 3-month follow-up (*P* < .001). In the intervention group, the intake of total energy and the percentage of energy from all fats, saturated fats, and trans fats decreased, and the intake of dietary fiber increased (all *P* < .01) postintervention.

**Conclusion:**

The worksite intervention improved metabolic and behavioral risk factors among employees with prediabetes. The long-term impact on diabetes prevention and program sustainability warrant further investigation.

## MEDSCAPE CME

Medscape, LLC is pleased to provide online continuing medical education (CME) for this journal article, allowing clinicians the opportunity to earn CME credit.

This activity has been planned and implemented in accordance with the Essential Areas and policies of the Accreditation Council for Continuing Medical Education through the joint sponsorship of Medscape, LLC and *Preventing Chronic Disease*. Medscape, LLC is accredited by the ACCME to provide continuing medical education for physicians.

Medscape, LLC designates this Journal-based CME activity for a maximum of 1 ***AMA PRA Category 1 Credit(s)™***. Physicians should claim only the credit commensurate with the extent of their participation in the activity.

All other clinicians completing this activity will be issued a certificate of participation. To participate in this journal CME activity: (1) review the learning objectives and author disclosures; (2) study the education content; (3) take the post-test with a 75% minimum passing score and complete the evaluation at www.medscape.org/journal/pcd; (4) view/print certificate.

**Release date: November 25, 2015; Expiration date: November 25, 2016**

### Learning Objectives

Upon completion of this activity, participants will be able to:

1. Describe the worksite lifestyle intervention used in this randomized trial among employees with prediabetes
2. Discuss the efficacy of a worksite lifestyle intervention for improving metabolic risk factors among employees with prediabetes, based on a randomized trial
3. Discuss the efficacy of a worksite lifestyle intervention for improving behavioral risk factors among employees with prediabetes

**EDITOR**

Ellen Taratus, Editor, *Preventing Chronic Disease*. Disclosure: Ellen Taratus has disclosed no relevant financial relationships.

**CME AUTHOR**

Laurie Barclay, MD, Freelance writer and reviewer, Medscape, LLC

Disclosure: Laurie Barclay, MD, has disclosed no relevant financial relationships.

**AUTHORS**

Kellie R. Weinhold, MS, RD, The Ohio State University, Columbus, Ohio

 Disclosure: Kellie R. Weinhold, MS, RD, has disclosed no relevant financil relationships.

 

Carla K. Miller, PhD, RD, The Ohio State University, Columbus, Ohio

Disclosure: Carla K. Miller, PhD, RD, has disclosed no relevant financial relationships.

 

David G. Marrero, PhD, Indiana University School of Medicine, Indianapolis, Indiana

Disclosure: David G. Marrero, PhD, has disclosed no relevant financial relationships.

 

Haikady N. Nagaraja, PhD, The Ohio State University, Columbus, Ohio

Disclosure: Haikady N. Nagaraja, PhD, has disclosed the following relevant financial relationships: Served as an advisor or consultant for: Abbott Nutrition.

 

Brian C. Focht, PhD, The Ohio State University, Columbus, Ohio

Disclosure: Brian C. Focht, PhD, has disclosed no relevant financial relationships.

 

Gregg M. Gascon, PhD, The Ohio State University Health Plan, Inc., Columbus, Ohio

Disclosure: Gregg M. Gascon, PhD, has disclosed no relevant financial relationships.

## Introduction

The prevalence of prediabetes among US adults increased significantly from 30.2% in 1999–2002 to 36.5% in 2007–2010 (1). People with prediabetes are at increased risk for type 2 diabetes mellitus (hereinafter referred to as type 2 diabetes) as well as microvascular and macrovascular comorbidities commonly associated with type 2 diabetes (2). Rising rates of prediabetes create an urgent need to prevent or delay the development of type 2 diabetes.

Intensive lifestyle interventions can prevent or delay type 2 diabetes in at-risk populations (3–5). These interventions target weight loss by improving dietary patterns and increasing physical activity (PA). The Diabetes Prevention Program (DPP), for example, demonstrated that weight loss through lifestyle modification was more effective than pharmacotherapy in reducing the incidence of type 2 diabetes among adults with prediabetes, and the relative reduction in incidence remained at 10-year follow-up (3,6). The current need is to translate effective interventions into practice settings.

Adults spend a large portion of their time at work, making the workplace a potentially effective setting for health promotion and disease prevention. Wellness programs can generate savings in medical costs and reduce absenteeism rates. Although worksite programs can promote weight loss (7), limited evidence exists on worksite diabetes prevention programs for employees at high risk of diabetes. The workplace could be an opportune setting for identifying employees with prediabetes, reducing risk through effective programs, and if employees avert type 2 diabetes, also reducing future costs (8).

The DPP has been adapted for community settings such as churches, hospitals, and YMCAs (9). Although the community-based programs promoted weight loss, thereby reducing risk for type 2 diabetes (3,6), few translational studies have evaluated the efficacy of worksite diabetes prevention programs (10–13). Among worksite studies that implemented the DPP, only 2 studies used a randomized design with a comparison condition (10,13), 2 studies reported the impact of the intervention on outcomes such as glucose and lipid levels (10,12), and none reported changes in dietary intake or objectively measured PA. Moreover, one worksite study implemented a primarily self-directed, low-intensity version of the DPP (13), limiting comparability; in addition, study attrition was high (11,13). The objective of our trial was to evaluate the efficacy of a worksite lifestyle intervention among employees with prediabetes. It was hypothesized that the intervention would facilitate greater reductions in the percentage of weight loss than would usual care.

## Methods

### Design

A randomized pretest–posttest control group design was used at a major university in the midwestern United States. Approval for the study was obtained from the university’s institutional review board, and all participants provided written, informed consent. Eligible participants were randomized at baseline to either a 16-week group-based lifestyle intervention or usual care from their health care providers (control condition) (Figure). Beginning in October 2012, participants were enrolled and allocated to treatment group in a 1-to-1 ratio by the project coordinator. Participants were randomized in blocks of 4 stratified by race and sex; 2 participants each were randomly assigned to the intervention or control arm. Assignment was generated by the statistician, and allocations were concealed in sequentially numbered opaque envelopes. The statistician was blinded to treatment assignment; however, neither participants nor lifestyle coaches were blinded to treatment allocation. All study appointments took place on the university campus. Postintervention data were collected after the 16-week intervention, followed by a 3-month maintenance period, during which participants received no contact from program staff; final data were collected 7 months after enrollment. Follow-up of participants was completed in May 2014 at trial end.

**Figure:**
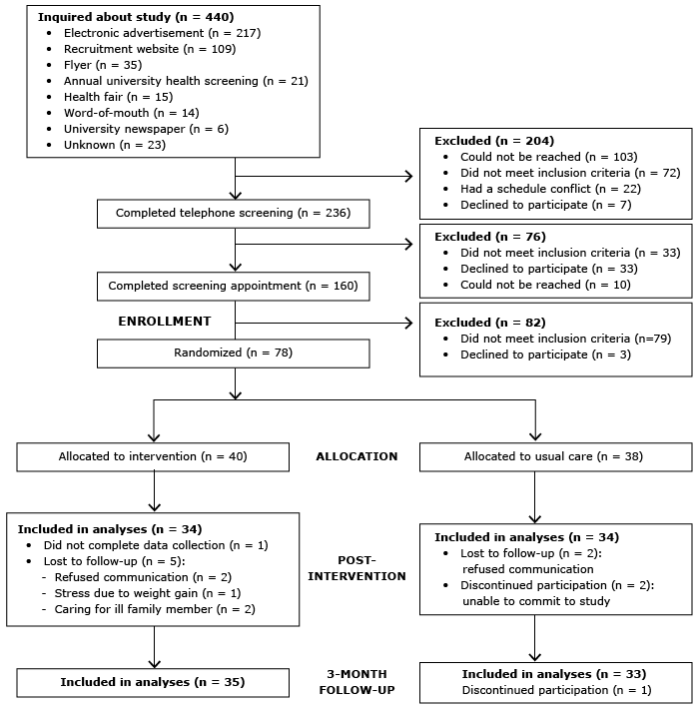
Phases of the randomized controlled trial for the intervention and usual care (control) groups in a university worksite diabetes prevention study, Ohio, 2012–2014.

### Sample

Potentially eligible participants with random glucose levels of 110 to 199 mg/dL during an annual employee health screening received a letter from the university health plan inviting them to participate. Additional study recruitment methods included campus flyers, electronic advertisements in digital newsletters, employee email notifications, and notices in ResearchMatch.com, a website for study recruitment.

Eligibility criteria included being an employee aged 18 to 65 and having a body mass index (BMI) of 25.0 to 50.0 kg/m^2^ and prediabetes. The 7-item American Diabetes Association risk assessment questionnaire was administered to determine risk for type 2 diabetes, and those with a score of 5 or more were classified as potentially eligible and screened for any exclusionary criteria (14). Eligible participants had fasting finger-stick glucose levels of 100 to 125 mg/dL, indicative of prediabetes (15). Individuals with fasting glucose levels of 95 to 99 mg/dL or 126 to 140 mg/dL completed a second finger stick to assess hemoglobin A1c (HbA1c); HbA1c levels of 5.7% to 6.4% indicated prediabetes and study eligibility (15). Individuals with HbA1c or glucose levels above the indicated ranges were advised to see their physician and excluded from participation.

Potentially eligible participants completed the Physical Activity Readiness Questionnaire; those who answered positively to 1 or more questions were excluded (16). Employees older than 65 receive a separate health plan through the state’s public employee retirement system; they do not routinely complete the biometric screening used for recruitment and were excluded from participation. Individuals were excluded if they had a current diagnosis of type 2 diabetes, were pursuing or recently had weight-loss surgery, were taking medications that modify blood glucose levels, such as metformin or corticosteroids, or were actively participating in a weight-management program. Participants could not be pregnant, breastfeeding, trying to become pregnant, or planning to leave university employment or move from the area within one year of study enrollment.

### Lifestyle intervention

Participants randomized to the intervention group received the manualized 16-week group-based intervention adapted from the DPP Outcomes Study Lifestyle Balance Program (17,18). The behavioral goals for the program were consistent with those of the DPP: achieve at least 7% weight loss, engage in at least 150 minutes per week of moderate to vigorous PA, and consume 25% or less of total energy from fat (3). The intervention focused on a lower-fat diet, and evidence suggests that reduced-calorie diets can produce clinically meaningful weight loss (19). Groups of 10 to15 participants met for 60 minutes weekly during the lunch hour or after work. Participants were encouraged to attend make-up sessions and obtain a weight measurement if they missed a session. Two university dietitians trained in intervention implementation served as lifestyle coaches for the weekly sessions. Written study materials, a book for estimating fat grams and calorie intake, and booklets for self-monitoring diet and PA were provided to participants. Participants received individual behavioral goals on weight, fat-gram intake, and PA during the first session, and they were encouraged to set small goals each week to facilitate progressive goal attainment. Participants engaged in group discussions and completed individual and group activities to foster skill development and social support. Participants were encouraged to self-monitor diet and PA daily and to submit monitoring booklets weekly for review by the lifestyle coach, who provided individualized feedback.

Participants in the control group received usual care from their health care providers. At baseline they received a booklet developed by the National Diabetes Education Program that describes strategies for self-regulated weight loss (20). Use of these materials is associated with modest weight loss (21). In their written materials, the control group was given a 7% target for weight loss. Following completion of all data collection, control group participants were invited to attend an informational session that addressed key weight-loss principles from the intervention.

A checklist for monitoring adherence to the intervention program was used to ensure fidelity. The principal investigator observed more than 20% of intervention sessions and found no serious departures from the curriculum as planned.

### Measures

All study measures were collected at baseline, postintervention (4 months), and at 3-month follow-up (7 months). The primary outcome was percentage weight change; secondary outcomes were anthropometric measures (height, waist circumference), clinical measures (glucose, blood lipids, blood pressure), dietary intake, and PA levels. Body weight was measured using a calibrated digital scale (Health-O-Meter Professional). Height was measured at baseline using a standing stadiometer (Perspective Enterprises). For height and weight measurements, participants wore light clothing and removed shoes. Waist circumference was assessed using the National Health and Nutrition Examination Survey protocol (22). Anthropometric measurements were collected twice per visit, and mean values were used for analyses.

Finger-stick blood samples were collected after a minimum 8-hour fast. The Alere Cholestech LDX System (23) was used to complete point-of-care analysis of the blood samples for glucose, total cholesterol, high-density lipoprotein (HDL) cholesterol, and triglycerides. Low-density lipoprotein (LDL) cholesterol was calculated using the Friedewald equation (24). The Alere Cholestech LDX System was certified as accurate and reproducible by the Centers for Disease Control and Prevention’s Lipid Standardization Program and Cholesterol Reference Method Laboratory Network (23). At each assessment, 2 systolic and 2 diastolic blood pressure readings were collected from seated participants with an Omron Healthcare 7-Series home blood pressure cuff, which meets the protocol criteria for validation standards of the European Society of Hypertension (25).

Dietary intake was estimated from the self-administered full-length Block 2005 Food Frequency Questionnaire (FFQ) (NutritionQuest). Portion sizes ranged from ¼ cup to 2 cups, and a food portion visual aid was provided. Nine response options on frequency of intake were included. PA was assessed using the Lifecorder Plus Accelerometer (Suzuken-Kenz, Inc), which estimates minutes spent in light, moderate, and vigorous PA. Participants were instructed to wear the accelerometer on their hip at the waistline for all waking hours on 7 consecutive days during each assessment.

### Analysis

Between-group differences in baseline demographic characteristics were assessed using Pearson χ^2^ tests or Fisher exact tests; differences in age were assessed using a 2-sample *t* test. We developed and validated mixed models in which participants nested within treatment groups were used as random effects, and treatment group, time, and their interaction were used as fixed effects. Outcome variables were assessed in the framework of these models using Student *t* tests within a repeated measures analysis of variance framework for between-group comparisons of mean values at baseline, and between-group and within-group change from baseline to postintervention and from baseline to 3-month follow-up. Outcome variables with residuals containing skewed distributions were modeled on the logarithmic scale to achieve approximately normal distributions. Relationships between postintervention weight change and intervention participation were assessed using Spearman correlations. Power analysis (power = 0.90, 2-tailed α = .05) for the primary outcome, percentage weight change, based on a previous DPP translational study, indicated that 25 participants in each treatment group were needed to detect a 4.04% difference between groups (21). All analyses were completed using SAS software package JMP version 10 (SAS Institute, Inc).

## Results

We found no significant differences in demographic characteristics between eligible individuals who enrolled in the study and those who declined enrollment, except for age (50.6 vs. 44.9 years, respectively; *P* = .005). We found no significant baseline differences in demographic characteristics between treatment groups, except for occupation (*P* = .01) (Table 1). Similarly, we found no significant difference between treatment groups at baseline for study outcomes, except for significantly lower diastolic blood pressure in the control than intervention group (*P* = .04) (Table 2). We found no significant differences in participant characteristics at baseline between those who did and those who did not complete the study (all *P* > .05).

**Table 1:** Demographic Characteristics of Participants in a Diabetes Prevention Study at a University Worksite, by Treatment Group at Baseline, 2012–2014^a^

Characteristic	Intervention (n = 35)	Control (n = 34)	*P* Value
**Age, mean (SD), y**	51.6 (9.5)	51.0 (8.1)	.50^b^
**Race**			
White	27 (77.1)	30 (88.2)	.34^c^
Asian or black	8 (22.9)	4 (11.8)	
**Ethnicity**			
Non-Hispanic	35 (100.0)	33 (97.1)	.49^c^
Hispanic	0	1 (2.9)	
**Sex**			
Male	7 (20.0)	7 (20.6)	>.99^c^
Female	28 (80.0)	27 (79.4)	
**Education**			
Less than bachelor’s degree	15 (42.9)	9 (26.5)	.32^d^
Bachelor’s degree	11 (31.4)	12 (35.3)	
Postgraduate degree	9 (25.7)	13 (38.2)	
**Employment status**			
Full-time employment	32 (91.4)	33 (97.1)	.61^c^
Part-time employment	3 (8.6)	1 (2.9)	
**Marital status**			
Married	24 (68.6)	26 (76.5)	.59^c^
Not married	11 (31.4)	8 (23.5)	
**Occupation type** e			
Professional	12 (35.3)	19 (55.9)	.01^d^
Clerical	10 (29.4)	13 (38.2)	
Other	12 (35.3)	2 (5.9)	
**Years at current job**			
1–5	13 (37.1)	11 (32.4)	.27^d^
6–10	13 (37.1)	7 (20.6)	
11–15	3 (8.6)	6 (17.7)	
≥16	6 (17.1)	10 (29.4)	
**Student status**			
Nonstudent	30 (85.7)	32 (94.1)	.22^d^
Full-time student	3 (8.6)	0	
Part-time student	2 (5.7)	2 (5.9)	
**Number of people in the household**			
1	5 (14.3)	2 (5.9)	.64^d^
2	17 (48.6)	16 (47.1)	
3	6 (17.1)	5 (14.7)	
4	5 (14.3)	9 (26.5)	
≥5	2 (5.7)	2 (5.9)	
**Annual household income^e^, $**			
20,000–39,999	8 (23.5)	3 (8.8)	.22^d^
40,000–59,999	4 (11.8)	4 (11.8)	
60,000–79,999	6 (17.7)	6 (17.6)	
80,000–99,999	9 (26.5)	6 (17.6)	
≥100,000	7 (20.6)	15 (44.1)	

a Values are number (percentage) unless otherwise indicated.

b Two-sample *t* test of between-group difference; *P* value < .05 was used for significance.

c Fisher exact test of between-group differences; *P* value < .05 was used for significance.

d Pearson χ^2^ test of between-group differences; *P* value < .05 was used for significance.

e One participant in intervention group did not provide this information.

**Table 2:** Changes in Anthropometric, Clinical, Dietary, and Physical Activity Outcomes of Participants in a Diabetes Prevention Study at a University Worksite, by Treatment Group at Baseline, 4 Months, and 7 Months, 2012–2014

Outcome	ANOVA Model Effects			Time^a^	Intervention, Mean (SE) (n = 35)^b^	Control, Mean (SE) (n = 34)^b^	*P* Value^c^
	Time	Group	Group × Time				
Weight change, %^d^	—	—	—	Baseline	—	—	—
				4 Months	−5.5 (0.6)	−0.4 (0.5)	<.001
				*P* value	<.001	.81	—
				7 Months	−5.2 (1.0)	−0.2 (0.7)	<.001
				*P* value	<.001	.80	—
Body weight, kg	<.001	.02	<.001	Baseline	95.4 (2.9)	101.9 (2.9)	.12
				4 Months	−5.1 (0.6)	−0.4 (0.6)	<.001
				*P* value	<.001^e^	.56	—
				7 Months	−4.9 (0.6)	−0.4 (0.6)	<.001
				*P* value	<.001	.58	—
Body mass index, kg/m^2^	<.001	.15	<.001	Baseline	35.0 (1.0)	35.9 (1.0)	.53
				4 Months	−1.9 (0.2)	−0.1 (0.2)	<.001
				*P* value	<.001	.63	—
				7 Months	−1.7 (0.2)	−0.1 (0.2)	<.001
				*P* value	<.001	.76	—
Waist circumference, cm	<.001	.04	.01	Baseline	107.2 (2.0)	110.8 (2.0)	.21
				4 Months	−4.8 (0.9)	−1.3 (0.9)	.007
				*P* value	<.001	.15	—
				7 Months	−5.1 (0.9)	−2.1 (0.9)	.02
				*P* value	<.001	.02^e^	—
Fasting glucose, mg/dL	<.001	.11	.05	Baseline	108.9 (1.8)	110.8 (1.9)	.52^f^
				4 Months	−8.6 (1.6)	−3.7 (1.6)	.02^f^
				*P* value	<.001	.02	—
				7 Months	−8.2 (1.5)	−7.5 (1.6)	.69^f^
				*P* value	<.001	<.001	—
Total cholesterol, mg/dL	.01	.42	.06	Baseline	194.7 (4.7)	197.8 (4.8)	.64
				4 Months	−11.3 (4.4)	−1.6 (4.4)	.12
				*P* value	.01	.72	—
				7 Months	5.7 (4.4)	0.4 (4.5)	.40
				*P* value	.19	.92	—
LDL cholesterol, mg/dL	.08	.34	.21	Baseline	112.6 (4.1)	114.5 (4.2)	.75
				4 Months	−9.1 (4.2)	−0.2 (4.2)	.13
				*P* value	.03	.96	—
				7 Months	2.1 (4.0)	1.6 (4.2)	.93
				*P* value	.61	.71	—
HDL cholesterol, mg/dL	.06	.40	.06	Baseline	50.9 (2.5)	50.5 (2.5)	.71^f^
				4 Months	−0.04 (1.4)	−0.3 (1.4)	.95^f^
				*P* value	.67	.76	—
				7 Months	4.1 (1.4)	−0.3 (1.4)	.04^f^
				*P* value	.002	.92	—
Triglycerides, mg/dL	.24	.98	.99	Baseline	166.1 (13.1)	163.0 (13.4)	.99^f^
				4 Months	−8.0 (9.3)	−4.8 (9.3)	.92^f^
				*P* value	.24	.31	—
				7 Months	−12.9 (9.1)	−3.0 (9.4)	.98^f^
				*P* value	.34	.34	—
Systolic blood pressure, mm Hg	.02	.85	.02	Baseline	128.8 (2.4)	124.4 (2.4)	.21
				4 Months	−8.3 (2.2)	−0.4 (2.2)	.01
				*P* value	<.001	.86	—
				7 Months	−6.3 (2.1)	0.4 (2.2)	.03
				*P* value	.004	.85	—
Diastolic blood pressure, mm Hg	<.001	.95	<.001	Baseline	90.9 (1.6)	86.2 (1.6)	.04
				4 Months	−8.5 (1.2)	−1.8 (1.3)	<.001
				*P* value	<.001	.16	—
				7 Months	−7.5 (1.2)	−0.6 (1.3)	<.001
				*P* value	<.001	.64	—
Total energy, kcal	<.001	.10	.13	Baseline	1,797 (117)	1,903 (120)	.26^f^
				4 Months	−424 (99)	−183 (101)	.12^f^
				*P* value	<.001	.13	—
				7 Months	−318 (97)	−309 (100)	.73^f^
				*P* value	.002	<.001	—
Protein, % of total energy	.56	.73	.11	Baseline	15.8 (0.4)	14.9 (0.4)	.13
				4 Months	−0.3 (0.4)	1.0 (0.4)	.05
				*P* value	.50	.03	—
				7 Months	−0.3 (0.4)	0.7 (0.4)	.12
				*P* value	.49	.13	—
Fat, % of energy	.30	.14	.007	Baseline	36.7 (1.0)	37.6 (1.1)	.54
				4 Months	−2.6 (0.8)	0.8 (0.9)	.008
				*P* value	.003	.39	—
				7 Months	−0.1 (0.8)	−0.2 (0.9)	.88
				*P* value	.94	.78	—
Saturated fat, g/1,000 kcal	.02	.09	.006	Baseline	12.6 (0.5)	13.3 (0.5)	.32
				4 Months	−1.5 (0.4)	−0.1 (0.4)	.01
				*P* value	<.001	.79	—
				7 Months	−0.3 (0.4)	−0.6 (0.4)	.57
				*P* value	.41	.11	—
Trans fat, g/1,000 kcal	.02	.11	.30	Baseline	1.3 (0.1)	1.4 (0.1)	.48
				4 Months	−0.2 (0.1)	−0.05 (0.1)	.13
				*P* value	.003	.42	—
				7 Months	−0.1 (0.06)	−0.1 (0.1)	.53
				*P* value	.04	.26	—
Monounsaturated fat, g/1,000 kcal	.63	.19	.03	Baseline	16.2 (0.5)	16.5 (0.5)	.67
				4 Months	−1.0 (0.5)	0.6 (0.5)	.02
				*P* value	.05	.19	—
				7 Months	0.2 (0.5)	0.2 (0.5)	.97
				*P* value	.73	.69	—
Polyunsaturated fat, g/1,000 kcal	.48	.70	.53	Baseline	8.9 (0.3)	8.8 (0.3)	.89
				4 Months	−0.17 (0.3)	0.3 (0.3)	.27
				*P* value	.59	.32	—
				7 Months	0.2 (0.3)	0.3 (0.3)	.75
				*P* value	.54	.30	—
Carbohydrate, % of total energy	.14	.31	.002	Baseline	46.7 (1.3)	46.8 (1.3)	.96
				4 Months	3.7 (1.0)	−1.0 (1.0)	<.001
				*P* value	<.001	.30	—
				7 Months	0.6 (1.0)	−0.04 (1.0)	.62
				*P* value	.51	.96	—
Fiber, g/1,000 kcal	<.001	.50	.06	Baseline	10.5 (0.6)	10.0 (0.6)	.87^f^
				4 Months	2.2 (0.5)	0.9 (0.5)	.05^f^
				*P* value	<.001	.06	—
				7 Months	0.8 (0.4)	1.2 (0.5)	.87^f^
				*P* value	.02	.02	—
Total sugars, g/1,000 kcal	.47	.34	.06	Baseline	51.6 (2.8)	52.5 (2.9)	.84
				4 Months	6.8 (2.8)	−2.4 (2.8)	.02
				*P* value	.02	.39	—
				7 Months	3.6 (2.7)	0.4 (2.8)	.41
				*P* value	.19	.90	—
Moderate/vigorous intensity physical activity, total minutes per week	.34	.28	.20	Baseline	122.6 (14.8)	106.9 (15.3)	.80^f^
				4 Months	23.9 (12.0)	2.2 (12.4)	.08^f^
				*P* value	.02	.83	—
				7 Months	6.8 (11.9)	9.9 (12.2)	.29^f^
				*P* value	.20	.80	—

Abbreviations: ANOVA, analysis of variance; HDL, high-density lipoprotein; LDL, low-density lipoprotein.

a Analyses conducted at baseline, change from baseline to postintervention (4 months), and change from baseline to 3-month follow-up (7 months).

b One participant in intervention group did not complete 4-month data collection, and 1 participant in control group did not complete 7-month data collection.

c Posthoc *t* test in a repeated measures ANOVA model for between-group comparison at baseline and to compare between-group change from baseline to 4 and 7 months; *P* value <.05 was used for significance.

d Analyses for primary outcome included student *t* test for within-group difference from 0 for the change at 4 and 7 months in intervention group; data were not normally distributed. Wilcoxon signed rank test was used for within-group difference from 0 for the change at 4 and 7 months in control group. *P* value <.025 was used for significance to account for the Bonferroni correction of multiple comparisons. One-way ANOVA compared between-group difference of means at 4 and 7 months; *P* value <.05 used for significance.

e Posthoc *t* test in a repeated measures ANOVA model to compare within-group change from baseline to 4 and 7 months. *P* <.025 was used for statistical significance to account for the Bonferroni correction of multiple comparisons.

f Variables were modeled on the logarithmic scale, and *P* values are for transformed data. Summary statistics are the original scale for data reporting.

We found a significant between-group difference in mean (SE) percentage weight change from baseline to postintervention (−5.5% [0.6], intervention; −0.4% [0.5], control; *P* < .001). Postintervention, the intervention group achieved significantly greater reductions in waist circumference, fasting glucose, and systolic and diastolic blood pressure than did the control group (all *P* < .025). Total cholesterol declined significantly (*P* = .01) in the intervention group.

The intervention group had a greater reduction in percentage energy from fat (*P* = .008) and a greater increase in fiber intake (*P* = .05) than the control group. In the intervention group postintervention, total energy intake decreased significantly (*P* < .001); intake of total fats, saturated fats, and trans fats decreased significantly; and carbohydrate and dietary fiber intake increased significantly (all *P* < .01).

Changes from baseline to 3-month follow-up remained significantly greater for the intervention than the control group for percentage weight change, body weight, waist circumference, and systolic and diastolic blood pressure (all *P* < .05). HDL cholesterol significantly increased from baseline to 3-month follow-up (*P* = .002) in the intervention group; this increase was significantly different from that of the control group (*P* = .04).

Changes in dietary intake and PA from baseline to 3-month follow-up were not significantly different between groups for any outcome. Total energy intake significantly decreased from baseline in the intervention group (*P* = .002) and control group (*P* < .001) at study end.

In the intervention group, 32.4% met the goal of achieving 7% or more weight loss postintervention, which was significantly more than in the control group (2.9%; *P* = .003). The proportion of participants in each group who lost more than 5% of their weight differed postintervention and at 3-month follow-up (Table 3). Fewer than 10% of participants in either group met the dietary goal of consuming 25% or less of total energy intake from fat at any time point. Postintervention, although few participants met the behavioral goal, fat intake had shifted from baseline more in the intervention than in the control group (*P* = .01).

**Table 3:** Attainment of Program Goals Among Intervention and Control Participants in a Diabetes Prevention Study at a University Worksite, by Treatment Group at Baseline, 4 Months, and 7 Months, 2012–2014^a^

Program Goal	Baseline			4 Months (Postintervention)			7 Months (3-Month Follow-Up)		
	Intervention (n = 35)	Control (n = 34)	*P* b	Intervention (n = 34)^c^	Control (n = 34)^c^	*P* b	Intervention (n = 35)	Control (n = 33)	*P* b
>5% Weight loss	—	—	—	18 (52.9)	1 (2.9)	<.001	17 (48.6)	3 (9.1)	<.001
0%–5% Weight loss	—	—	—	14 (41.2)	17 (50.0)		13 (37.1)	12 (36.4)	
Weight gain	—	—	—	2 (5.9)	16 (47.1)		5 (14.3)	18 (54.5)	
150 Min/wk MVPA	10 (28.6)	8 (23.5)	.79^d^	17 (50.0)	8 (25.0)	.05^d^	12 (34.3)	9 (27.3)	.60^d^
<30% Energy from fat	3 (8.6)	2 (5.9)	.87	13 (39.4)	3 (9.4)	.01	6 (17.1)	1 (3.0)	.13
30%–35% Energy from fat	8 (22.9)	7 (20.6)		6 (18.2)	5 (15.6)		6 (17.1)	9 (27.3)	
>35% Energy from fat	24 (68.6)	25 (73.5)		14 (42.4)	24 (75.0)		23 (65.7)	23 (69.7)	

Abbreviations: MVPA, moderate to vigorous intensity physical activity.

a Values are number (percentage) unless otherwise indicated

b Pearson χ^2^ test of between-group differences in proportions; *P* value <.05 was used for significance.

c One participant in intervention and 2 in control group did not complete dietary questionnaire postintervention; 2 participants were missing accelerometer data in the control group postintervention.

d Fisher exact test of between-group differences in proportions; *P* value <.05 was used for significance.

The mean (standard deviation [SD]) number of sessions attended by intervention participants was 11.6 (4.5) or 72.5% of the 16 sessions offered. Participants recorded their dietary intake for a mean (SD) of 5.6 (1.5) days per week, and reported a mean (SD) of 161.9 (135.0) minutes per week of PA. In the intervention group, the change in body weight postintervention was negatively associated with total days of self-monitoring dietary intake (*r* = −0.62, *P* < .001), total minutes spent in PA (*r* = −0.49, *P* = .004), and total number of sessions attended (*r* = −0.60, *P* < .001).

## Discussion

The 16-week group-based lifestyle intervention delivered at a university worksite facilitated reductions in body weight among employees with prediabetes. These results support the efficacy of the intervention for promoting risk reduction (3) and provide evidence for its utility at the worksite. Whereas 50% of participants who received the original 24-week DPP intervention, which included individual counseling, lost at least 7% of body weight (3), 32.4% of intervention participants in our study lost that amount. The smaller proportion in our study may have resulted from the shorter intervention period and group delivery format. We chose a group delivery format for a worksite approach to reduce personnel demands and enhance employee cohesion and support.

Weight loss in the DPP was the primary predictor of reduction in diabetes incidence; every percentage point of weight loss achieved at the end of the intervention contributed to a 10% reduction in diabetes risk, independent of glycemic changes (26). Mean postintervention weight loss in our study was 5.5%, which is considered clinically significant and is frequently associated with reduced morbidity and mortality (27). An 8-year longitudinal study examined changes in risk factors after a worksite wellness program at a US university and found that obese participants who achieved and maintained a BMI less than 30 kg/m^2^ for 3 consecutive years reduced the risk of developing type 2 diabetes by 78% compared with those who remained obese (28). Thus, a mean weight loss of 5.5% following the intervention in our study contributes to risk reduction for type 2 diabetes. Long-term follow-up is needed to determine the progression to type 2 diabetes after weight loss.

Fasting glucose values returned to near-normal levels in the intervention group postintervention, and the improvement was maintained at 3-month follow-up. Each 5 mg/dL decrement of fasting glucose at 6 months in the DPP was related to a 26% reduction in diabetes risk (26). We did not assess postload glucose. DPP reports identified the lifestyle intervention as being superior to metformin in restoring normal postload glucose values; however, that was not the case for fasting glucose (3). Further research of worksite translations of the DPP should consider assessing both fasting and postload glucose; however, the assessment of postload glucose increases participant burden and study costs.

PA levels in the intervention group approached the goal of 150 minutes per week postintervention. However, PA returned to near baseline levels after the 3-month maintenance phase. Half of the intervention group achieved the PA goal postintervention, suggesting that this population of primarily working women struggled to initiate and sustain regular PA. The postintervention decline in PA is consistent with findings from other lifestyle interventions documenting that overweight individuals with or at risk for chronic disease often rapidly return to sedentary behavior upon intervention completion (29). However, our findings are in contrast to the DPP, in which 74% of lifestyle participants met the goal immediately after intervention completion, and 67% did 2 years later (30). Working adults may need more support than the general population to increase PA levels. Incentives used in the DPP to keep participants at goal may have been responsible for the higher levels of PA observed (18). Free-of-charge membership to campus exercise facilities might enhance engagement in PA for university worksite studies. A meta-analysis of PA interventions concluded that to be most effective, interventions targeting PA should include self-monitoring, goal setting, and modeling PA behaviors (31). Although our intervention included both PA self-monitoring and goal setting, the findings suggest that greater emphasis on the development and practice of key PA-related self-regulatory skills (ie, goal setting, barrier problem solving, PA planning) necessary to facilitate the transition to independent maintenance of PA may also promote long-term PA adherence.

Study participants reported changes in dietary intake. Total energy intake and percentage energy from fat decreased and fiber intake increased significantly in the intervention group postintervention. Lower fat intake and greater fiber intake in combination with modest weight loss and increased PA was associated with reduced risk for type 2 diabetes among at-risk adults (32,33). Reducing fat intake to less than 30% of energy is associated with preventing weight regain after periods of energy restriction, highlighting the benefits of reduced fat intake for long-term maintenance of weight loss (34).

Energy intake at 3-month follow-up also decreased significantly (*P* < .001) in the control group. All eligible participants were informed after screening that their glucose values were in the prediabetes range, and the control group received written materials on diet and PA changes to promote weight loss. Knowledge of their own prediabetes may promote behavioral change in at-risk individuals. Additionally, participants may have reported lifestyle changes because of repeated assessment of weight, diet, and PA behaviors, consistent with the Hawthorne effect.

Although results from our study are promising, some limitations exist. Follow-up was limited to 3-months postintervention; thus, long-term outcomes are unknown. A larger translational study with longer follow-up is needed to further evaluate effectiveness in delaying or preventing type 2 diabetes. A worksite approach is promising for tracking disease progression, especially when annual employee biometric screenings are implemented. For worksite adoption, the lifestyle intervention needs to be cost-effective, sustainable, and compatible with existing health care systems. Future research should evaluate the cost-effectiveness and long-term sustainability of worksite translations of the DPP. In our study, 80% of the participants were women, whereas 62% of the benefit-eligible university employees were women. Disparity in enrollment by sex is common in weight-loss studies, and recruitment of men deserves further evaluation. Men may benefit from Internet-based gender-tailored programs (35). Future research should address how best to tailor programs to the interests and needs of men and racial/ethnic minority populations.

Prediabetes is a growing problem in the United States and places individuals at increased risk for type 2 diabetes. Worksites can be effective settings for offering health promotion programs to employees; however, few studies have evaluated the impact of the DPP intervention at the worksite. Our study contributes to the limited evidence and demonstrates, through a randomized design, the feasibility and efficacy of the group-based DPP intervention in facilitating improvement in lifestyle behaviors, weight control, and reduction in metabolic risk for type 2 diabetes among university employees with prediabetes.

## Figures and Tables

**Table:** 

**1. The activity supported the learning objectives.**				
**Strongly Disagree**				**Strongly Agree**
1	2	3	4	5
**2. The material was organized clearly for learning to occur.**				
**Strongly Disagree**				**Strongly Agree**
1	2	3	4	5
**3. The content learned from this activity will impact my practice.**				
**Strongly Disagree**				**Strongly Agree**
1	2	3	4	5
**4. The activity was presented objectively and free of commercial bias.**				
**Strongly Disagree**				**Strongly Agree**
1	2	3	4	5

## Post-Test Information

To obtain credit, you should first read the journal article. After reading the article, you should be able to answer the following, related, multiple-choice questions. To complete the questions (with a minimum 75% passing score) and earn continuing medical education (CME) credit, please go to http://www.medscape.org/journal/pcd. Credit cannot be obtained for tests completed on paper, although you may use the worksheet below to keep a record of your answers. You must be a registered user on Medscape.org. If you are not registered on Medscape.org, please click on the "Register" link on the right hand side of the website to register. Only one answer is correct for each question. Once you successfully answer all post-test questions you will be able to view and/or print your certificate. For questions regarding the content of this activity, contact the accredited provider, CME@medscape.net. For technical assistance, contact CME@webmd.net. American Medical Association's Physician's Recognition Award (AMA PRA) credits are accepted in the US as evidence of participation in CME activities. For further information on this award, please refer to http://www.ama-assn.org/ama/pub/about-ama/awards/ama-physicians-recognition-award.page. The AMA has determined that physicians not licensed in the US who participate in this CME activity are eligible for ***AMA PRA Category 1 Credits*** ™. Through agreements that the AMA has made with agencies in some countries, AMA PRA credit may be acceptable as evidence of participation in CME activities. If you are not licensed in the US, please complete the questions online, print the AMA PRA CME credit certificate and present it to your national medical association for review.

## Post-Test Questions

### Article Title: A Randomized Controlled Trial Translating the Diabetes Prevention Program to a University Worksite, Ohio, 2012–2014

**CME Questions**

1. You are consulting for a large health maintenance organization regarding use of a worksite lifestyle intervention for employees with prediabetes. Which of the following statements about the worksite lifestyle intervention used in the randomized trial by Weinhold and colleagues is ***correct***?
  - The intervention was adapted from the Diabetes Prevention Program Outcomes Study Lifestyle Balance Program
  - The intervention focused on a lower-carbohydrate diet
  - Groups of 10 to 15 participants met for 30 minutes weekly for 12 weeks during the lunch hour or in the evening after work
  - Participants received daily diet and physical activity self-monitoring booklets and submitted them daily for review by the lifestyle coach
2. According to the randomized trial by Weinhold and colleagues, which of the following statements about the efficacy of a worksite lifestyle intervention for improving metabolic risk factors among employees with prediabetes is ***correct***?
  - Mean weight loss was 3% in the intervention group and 2% in the control group after the intervention
  - Weight loss in the intervention group was not sustained at 3-month follow-up
  - Mean fasting glucose reductions were not significantly greater in the intervention group than in the control group
  - Both groups had significant glucose reductions at 3-month follow-up (P <.001)
3. According to the randomized trial by Weinhold and colleagues, which of the following statements about the efficacy of a worksite lifestyle intervention for improving behavioral risk factors among employees with prediabetes would ***most*** likely be correct?
  - Postintervention, total energy and percent energy from total, saturated, and trans fats did not decrease significantly in the intervention group
  - Postintervention, dietary fiber did not increase significantly in the intervention group
  - Behavioral goals were to achieve at least 7% weight loss, obtain at least 150 minutes per week of moderate to vigorous physical activity, and consume 25% or less of total energy from fat
  - The study proves that the workplace intervention improves behavioral risk factors and sustains improvement over the long-term

**Evaluation**
